# The effect of emotional arousal on visual attentional performance: a systematic review

**DOI:** 10.1007/s00426-023-01852-6

**Published:** 2023-07-07

**Authors:** Andras N. Zsidó

**Affiliations:** Institute of Psychology, University of Pécs, 6 Ifjusag Str., Pécs, 7624 Hungary

## Abstract

Although the arousal elicited by emotional stimuli, similarly to valence, is an integrative part of emotion theories, previous studies and reviews mostly focused on the valence of a stimulus and rarely investigated the role of arousal. Here, I systematically searched for articles that used visual attentional paradigms, manipulated emotional arousal by auditory or visual, task-relevant or task-irrelevant stimuli, measured behavioral responses, ocular behavior, or neural correlates. I found that task-relevant arousing stimuli draw and hold attention regardless of the modality. In contrast, task-irrelevant arousing stimuli impaired task performance. However, when the emotional content precedes the task or it is presented for a longer duration, arousal increased performance. Future directions on how research could address the remaining questions are discussed.

## Introduction

The present review was primarily motivated by a desire to address confusion regarding the unique role of arousal through the visual search literature, and to emphasize the importance of using precise and uniform terminology. As Reisenzein pointed out “*the quality of an emotion depends on the proportion of pleasure-displeasure and arousal (or activation-deactivation) that is experienced*” (Reisenzein, 1994, p. 527). Emotional arousal has received little attention in previous research, although it is an integrative and equally important part of dimensional models of emotions along with valence (Bliss-Moreau et al., 2020; Bradley et al., 1992; Feldman Barrett & Russell, 1998; Rubin & Talarico, 2009; Watson et al., 1999). However, this trend seems to change in recent years (Onie & Most, 2021; Singh & Sunny, 2017), prompting the need for a systematic review of past research that could aid future studies in answering the most burning questions. A meta-analysis (Pool et al., 2016) of attentional biases for positive emotional stimuli underscores the importance of arousal by noting that the magnitude of effects is moderated by the arousal level of the stimuli used. The fact that previous research investigating the interaction between emotion and visual attention mainly focused on the effects of the valence of a stimulus (i.e., positive or negative) on various attentional processes, and, thus, often neglected the role of arousal (Yiend, 2010) might not be evident at first. This is mostly due to a major confound in the field: the terminology is not used in a precise and consistent way, and, thus, the two dimensions are conflated. In fact, some previous studies claim to have found an effect of emotionally arousing stimuli, whereas they used positively and negatively valenced stimuli and did not directly manipulate the arousal level of the stimuli used. In the present article, the primary intention is to capture papers where the effect of the arousal dimension of the stimuli was directly manipulated and empirically tested.

Emotional arousal can be defined as a state of physiological activation; heightened levels evoke feelings of excitement, nervousness, or alertness—creating a readiness for action—while decreased levels induce relaxation or boredom (Mather & Sutherland, 2009; Pessoa, 2013; Reisenzein, 1994). Further, emotional arousal can be described as the degree of subjective activation an observer experiences when viewing a stimulus (Pessoa, 2013; Reisenzein, 1994). There is a duality in the definition of emotional arousal concerning the subjective and objective nature of arousal which is also evident in its measurement. On the one hand, subjective (emotional) arousal is often measured by self-report questions (e.g., How aroused you felt while viewing the picture? Rate on a 9-point Likert-type scale where 1 means Calm and 9 means Excited.) asking the participant to report the feelings that an emotionally charged stimulus elicited (Lang et al., 1997). On the other hand, objective (emotional) arousal can be shown by various physiological changes such as increased skin conductance (Cuthbert et al., 2000; Kasos et al., 2019), decreasing nose-tip temperature (Sato et al., 2020), and greater N1, P2, P3 event-related potential amplitudes (Olofsson et al., 2008). Nonetheless, previous studies found a strong correlation between subjective and objective arousal measures (Cuthbert et al., 2000; Kasos et al., 2019; Olofsson et al., 2008; Sato et al., 2020). Still, with arousal (similarly to other emotion-related variables) it is a question if one can be sure to have measured it. Previous studies examining various effects of arousal usually take on the same approach, that is, they use a mix of positively and negatively valenced stimuli that are considered highly arousing (e.g., pictures, music, facial expressions). A correlation between subjective and objective measures is also a validating factor. Since both subjective feelings and objective changes count, one can mostly be sure to have measured arousal if they point in the same direction.

Stimuli with emotional content (e.g., pictures, music, words) initiate generalized arousal in the organism by releasing biogenic amines, such as norepinephrine, and hormones, such as cortisol (LeDoux, 2012). That is, arousal conveyed by the stimulus could initiate a readiness for action due to a heightened state of physiological activation (Bradley et al., 2008; Olofsson et al., 2008; Pessoa, 2013). Further, arousal may enhance processing in the survival circuit—a hardwired, evolutionarily conserved set of defensive mechanisms activated in challenging and possibly life-threatening situations—which facilitates activity in sensory, memory, and attentional processes (LeDoux, 2012, 2022). However, extreme levels of subjective arousal could lead to worse performance compared to lower levels of arousal because the available cognitive capacity is restricted by the arousal level that competes with task-relevant processes (Curci et al., 2013; Kahneman, 1973; Plass & Kalyuga, 2019; Schmeichel, 2007). Further, arousal—if not task-relevant—can also be distracting because stimuli that evoke a higher level of arousal may be prioritized over less arousing stimuli in the competition for processing (Buodo et al., 2002; Mather & Sutherland, 2011; Unsworth & Robison, 2017). In the past 50 years, a few theories were coined to describe the effect of arousal (independently of valence) on visual cognition. In the present article, by collecting the results of visual attention-related studies, I also aim to compare which theory (if any) can best describe the range of findings.

### Theories of arousal and attention

The first theory that clearly outlines the potential effects of arousal on cognitive, and in particular attentional, processing is the *limited capacity of attention* theory (Kahneman, 1973). Although this theory is mainly known as the first to describe attention as an effort and in terms of working memory capacity as opposed to the then-dominant filter models, the level of subjective arousal has a crucial role in determining which stimuli are processed. This is because according to the *limited capacity of attention* theory, the attentional (and working memory) capacity is arousal-dependent. That is, to some extent the actual level of arousal positively predicts what proportion of the total capacity will be available. Presumably, the most well-described theory of how arousal affects attention is the *arousal-bias competition* theory (Mather & Sutherland, 2011). This theory states that arousal can amplify the effects of contrast in bottom-up processes and prioritize the processing of a stimulus through top-down processes. That is, the priority of emotional stimuli in attentional processing may be due to the arousal level such stimuli convey (Sutherland & Mather, 2018).

There are several possible explanations for the background mechanisms of *arousal-bias competition* regarding visual attention performance. One of these explanations (1) is offered by the *glutamate amplifies noradrenergic effect* (Mather et al., 2016). The authors argue that prioritization can be possible through the locus coeruleus-norepinephrine system that amplifies the activation of prioritized stimuli while also inhibiting the activation of other (nonprioritized) stimuli (Mather et al., 2016). Hence, the most arousing stimuli would be favored in the competition for attentional allocation and will gain access to available cognitive resources first. Another explanation (2) could be that the arousing stimuli can gain access to the shortcut offered by the *superior colliculus-pulvinar-amygdala pathway* (Pessoa & Adolphs, 2010). This is the core of the brainstem–amygdala–cortical alarm system (Csathó et al., 2008; Koller et al., 2019; Liddell et al., 2005) which was initially described to account for the quick and accurate detection of threatening stimuli under disadvantageous conditions (e.g. non-conscious detection or unseen stimuli). The *superior colliculus-pulvinar-amygdala pathway* theory postulates that the amygdala has a key role in selectively processing those inputs that are the most relevant to the goals of the perceiver. This is consistent with appraisal theories positing that organisms have evolved to quickly detect stimuli that are relevant to their current needs (Brosch et al., 2013; Sander et al., 2005, 2018). Concerning the role of arousal, a meta-analysis (Pool et al., 2016) showed that attentional biases are more pronounced when the emotional stimulus is relevant to the perceiver (e.g., a hungry person seeing a picture of food). Although this means a somewhat broader perspective and is not exclusive to the arousal level of the stimulus, the *superior colliculus-pulvinar-amygdala pathway* theory often cites features (e.g., salience, significance, ambiguity, unpredictability) that have higher levels of elicited arousal in common. Further, the amygdala is also thought to allocate processing resources to stimuli which leads us to a theory that has a different take on the role of arousal. The third explanation (3)—the *arousal stimulation effect*—builds on both the *limited capacity of attention* and *arousal-bias competition* theories. According to this, the arousal level conveyed by the stimuli can facilitate overall attentional performance by speeding the movement of attention over time and increasing the available capacity devoted to the task (2019b; Zsido, et al., 2018). Further, the *arousal stimulation effect* also has a mutual point with the *superior colliculus-pulvinar-amygdala pathway*, that is, both emphasize the role of the processing speed of the cortex (and the availability of the resources) which might make a swift subcortical route redundant.

The *common points* of these arousal theories are that (1) working memory has a limited capacity for ongoing visual processes (Bundesen, 1990; Fukuda et al., 2010; Luck & Vogel, 2013), (2) the increased arousal level increases activity in the central arousal system such, e.g. the locus coeruleus norepinephrine system (Howells et al., 2012) that arise from the brainstem (Moruzzi & Magoun, 1949), and (3) higher levels of arousal conveyed by threatening stimuli can (seemingly) increase the capacity limit of working memory. There are *two core differences* in theories: (1) whether they accept that the increase in the capacity limit of working memory is real or discard this and argue that it is just apparent and (2) how they explain the apparent increase. Nevertheless, it seems plausible to claim that the effect of emotional arousal can be as crucial on cognition, and attentional processes in particular, as that of emotional valence. Studies utilizing neurological assessment methodologies, such as EEG and fMRI, may help disentangle the background mechanisms of the impact emotional arousal has on attentional processes. Previous EEG studies (Hajcak et al., 2010; Schupp et al., 2007) identified three key components of the event-related potential (ERP) that were consistently linked to the processing of emotional stimuli. (1) The early posterior negativity (EPN, 200–300 ms), a highly automatic component that has to do with selective visual attention (“Watch out something relevant is there!”), studies typically find an increased EPN for emotional stimuli (Schindler & Kissler, 2016). (2) The P300 (250–500 ms) is an important signature of cognitive processes such as attention and working memory, a neural response to stimulus significance, a larger amplitude can be observed for more important events (Hajcak & Foti, 2020). (3) The late positive potential (LPP, beginning around 400–500 ms) is associated with the rapid and dynamic allocation of increased attention to emotional stimuli and is also sensitive to the arousal level of the stimuli (Hajcak et al., 2009). A large body of neuroimaging studies underscores the role of the amygdala and ascending modulatory neurotransmitter system in emotional processing (LeDoux & Daw, 2018; Phelps et al., 2004; Vuilleumier, 2015). In the present review, my goal was to investigate the effects of arousal on visual attentional performance. Further, to gather evidence on the importance of taking emotional arousal into account in future experiments on the interplay of emotions and visual attention.

### Arousal-induced differences in feature-based and spatial theories of attention

Underlying attentional mechanisms behind visual processing are still somewhat polarized based on whether they apply a feature- or object-based (hereinafter, I treat these as one category for the sake of simplicity) or a spatial model. Feature-based model theories, in general, emphasize *selection based on features* or whole objects irrespective of spatial information and use tasks such as visual search or attentional blink (Mozer & Vecera, 2005). That is, for instance, the inputs from a visual scene compete for access to the working memory and, thus, further processing (Desimone & Duncan, 1995). In visual search tasks (VST) participants have to find a previously defined target image (e.g. a snake) among distractor images of a different category (e.g. flowers). The idea is that the more salient an image is (such as an emotionally charged one), the more it will draw participants’ attention. When assessing the attentional bias associated with emotional stimuli, the target is either an emotionally charged or a neutral image, and the distractors are of the other category (i.e., neutral or emotionally charged images, respectively). Performance is measured by the reaction time (RT) of finding the target and in some cases by accuracy. For attentional blink paradigms (AB), the rapid serial visual presentation (RSVP) technique is used to present the stimuli, where images follow each other in rapid (typically 50–200 ms, i.e., 5–20 items per second) succession and the task is to detect if a target is present (typically it is in 50% of the trials) in the stream and then to report whether a second target also occurred. AB occurs when the first target is correctly identified but then the participant fails to report the presence of the second target. Here, emotionally charged stimuli should be less affected by AB compared to neutral ones because they are more salient. Performance is measured by the accuracy of finding the second target. All these tasks can use task-irrelevant emotional stimuli, e.g., flashing up such images before or instead of the fixation cross before the start of the trials or playing music in the background that triggers an affective response.

In *spatial theories,* the main property for stimulus selection (and, later, feature binding) is the location of the stimulus (Mozer & Vecera, 2005). Based on the work of Petersen and Posner (Petersen & Posner, 2012; Posner & Petersen, 1990) such experiments examine how emotionally charged stimuli are processed by the attentional networks (alerting, orienting, and executive). Studies building on a spatial theory tend to use spatial cueing paradigms (e.g., dot-probe or spatial cueing task), free viewing, or Attention Network Task (ANT). In the spatial cueing tasks, emotionally charged and neutral stimuli are presented (typically onset for 100–1000 ms) randomly on either side of the screen. The images are then preceded by a probe (a target, e.g.: and a foil, e.g.,) and the participant has to indicate the side the target appeared. When the target appears in the position of the emotionally charged image, participants are expected to respond faster compared to when the target is presented at the location of the neutral image. Performance is measured by RT of finding the target and in some cases also by accuracy. In free viewing tasks participants have to explore images presented one at a time or multiple ones in an array. Here, the dwelling time, i.e. time spent looking at a given part of an image or one image compared to others presented can be measured. Dwelling time is expected to be longer for emotionally charged images. The ANT usually consists of three tasks accessing all three attentional networks in the Posner-Petersen model. The alerting network is examined by responding to a warning signal and measuring changes in RT. The orienting network is assessed by a spatial cuing task. The executive network is examined by a flanker task. In the flanker task, participants have to indicate the direction (left or right) of a central arrow surrounded by congruent, incongruent or neutral flankers (arrows pointing in the same or different direction, respectively). Both RT and accuracy can be measured. Similarly to the feature-based ones, all these tasks have the possibility to use task-irrelevant emotional stimuli, by e.g., presenting emotional pictures as non-relevant distractors during a task or playing music in the background.

### Task relevance of the emotionally arousing stimuli

Regarding emotionally charged stimuli in general, it has long been argued (Calvo & Castillo, 2005; Cisler & Koster, 2010; McNally, 2018; Quinlan, 2013; Richards et al., 2012; Subra et al., 2017) that to fully understand their effects using only *task-relevant* stimuli is not feasible. In such experiments, the emotional stimuli are central to the task, i.e. participants are asked to process them to correctly complete the task. Therefore the results are mostly indicative of the effects on goal-directed attentional processes but not stimulus-driven ones (Corbetta & Shulman, 2002; Subra et al., 2017). Furthermore, since top-down processes (searching strategy and task demands) greatly determine performance, unintentional or automatic processes (e.g., attentional capture) cannot be measured (Frischen et al., 2008; Yantis, 1993). To overcome this limitation, some studies started using *task-irrelevant* emotionally charged images (e.g., as distractors presented during a visual search task). Thus, in the present review, I sought to investigate the effects of arousal separately when task-relevant and task-irrelevant stimuli were used to understand how arousal affects goal-directed and stimulus-driven attentional processes.

### Goals of the current review

I have often found an arousal-related effect in my previous studies but found it difficult to compare it with other studies because they rarely manipulated arousal levels or the terminology they used was misleading. Previous reviews were mostly concerned with the effects of the valence of emotionally charged stimuli (i.e. positive, negative, neutral) on visual processing (Gerdes et al., 2014; Olofsson et al., 2008). Therefore, the purpose of the present systematic review is to review the results of previous studies dealing with the effects of emotional arousal on visual attentional performance. I aimed to fill a gap as, to my knowledge, to date, there is no publication available that systematically reviews the effects of emotional arousal on visual attentional performance. In this paper, I review how emotional arousal conveyed by task-relevant and irrelevant stimuli affects visual attentional performance indicated by visual search tasks. I hope that this review may encourage future research to further examine the effects of (emotional) arousal not just in visual attention-related processes but in other areas of cognition as well.

## Methods

I have prepared this systematic review in accordance with the recommended reporting items for systematic review and meta-analysis protocols (PRISMA) 2020 checklist to include in a systematic review protocol (Page et al., 2021). I used a two-stage systematic approach to identify articles that examined the effect of emotional arousal on visual search performance. The initial search was conducted on 1 March 2022 in the Web of Science database using the following combinations of keywords: ("*emotion*" OR "*emotional*") AND ("*arousal*") AND ("*visual attention*" or "*visual search*" OR "*visual detection*") with (“*participants*” OR “*subjects*” OR “*patients*”). The following predetermined criteria were necessary for further inclusion:

1. Scholarly (peer-reviewed) journal article
2. Empirical study (no reviews or meta-analyses)
3. Adult (18 + years) human participants
4. Visual attentional performance was investigated
5. The purpose was to examine the effect of emotional arousal
6. Emotional stimuli must have been used in the paper/study to elicit arousal

No restrictions were made concerning the modality of the emotional stimuli (e.g., auditory, visual), task or paradigm and methodology (e.g., behavioral, EEG, fMRI) used, and mental or physical health of the participants. After the initial search, I removed duplicates and examined the resulting articles’ references to ensure all relevant papers were included. I did not analyze effect sizes due to differences in the type of tasks, emotional stimuli, and timing methods used.

## Results

**Fig. 1:**
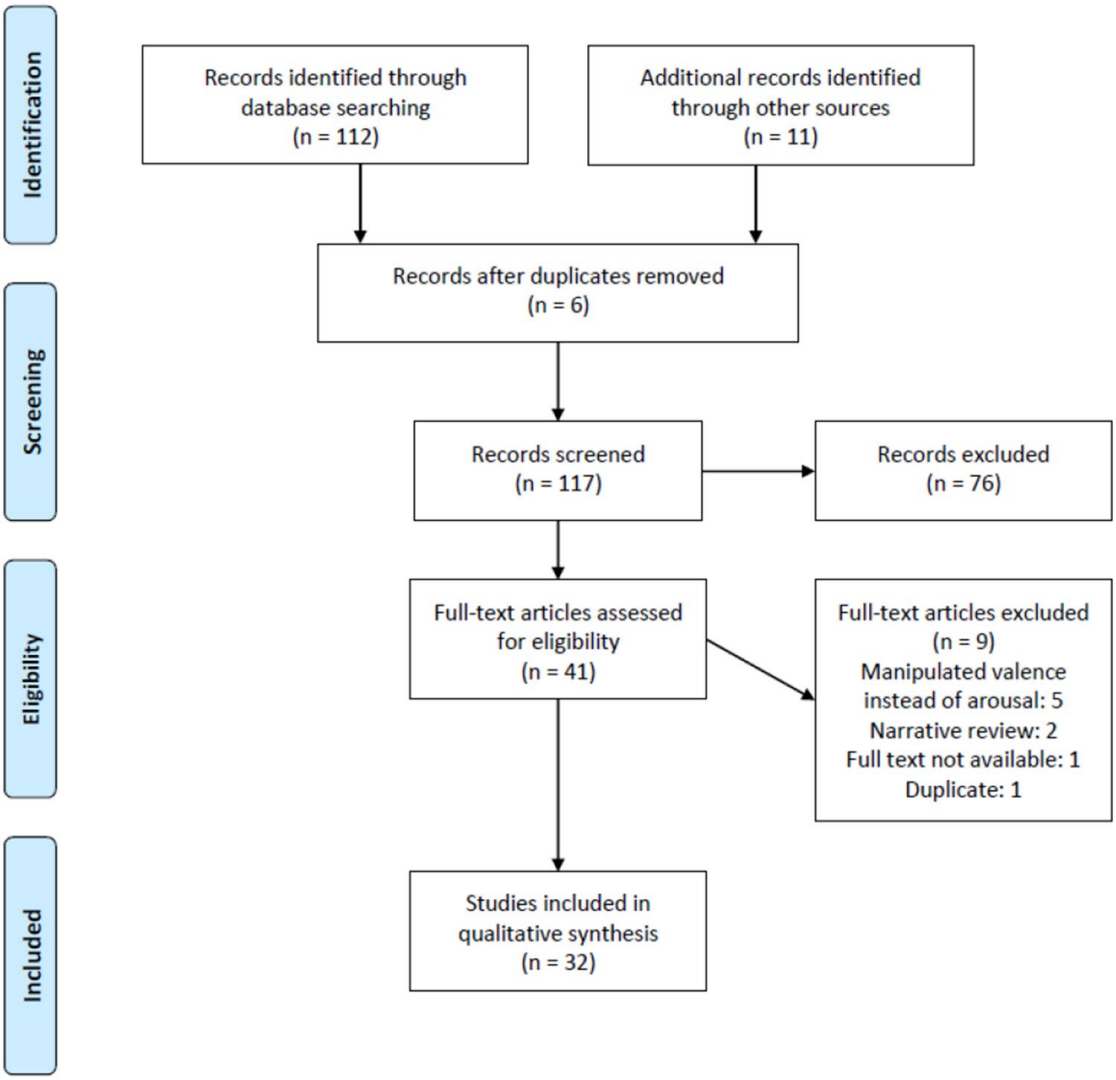
Flow diagram of the selection and review process for review articles

The search yielded a total of 123 articles published between 2000 and 2022 of which 32 articles fit the search criteria. A summary of our search strategy is presented in the Preferred Reporting Items for Systematic Reviews and Meta-Analyses (PRISMA) Flow Diagram (see Fig. 1). A summary of each article is presented in Table 1. The papers contain a total of 46 experiments with an average sample size of 49.78 and an average female-to-male ratio^1^ of 0.60. The modality of the stimuli used in the studies was not always similar across experiments reported, therefore, here I report the type of stimuli per experiment. A total of 38 experiments used visual stimuli (29 used pictures, seven used emotion-laden words, and one used both), while eight studies used auditory stimuli in addition to visual stimuli (six used sounds, and two used classical music). The majority of the experiments are exclusively behavioral (34), while five studies measured EEG, five studies used an eye-tracking device, and one study included fMRI measures. Of all the papers, the majority (15) used a visual search task (in 19 experiments), three studies used an attentional blink task (in eight experiments), three studies used spatial cueing (in four experiments), three studies used free viewing (in four experiments), one study used a pair-matching task (in one experiment), and one study used ANT (in one experiment). Six additional studies used other paradigms where the emotional stimuli were not central to the task (they were task-irrelevant) and had to be ignored. These studies used the number matrix paradigm (six experiments), solving arithmetic operations (three experiments), target counting (one experiment), or locating other targets (one experiment) as the main task. Furthermore, regarding stimuli selection studies relied on standardized arousal ratings provided by the manuals of the stimuli database. In most cases, there was no further validation, while in some instances the stimuli presented in the experiment were also rated by an independent sample or by the participants in the study. In one study, the was validated by fMRI results with the difference between BOLD responses (see Table 1 for the details). In the results section, I present the results of the papers in three subsections: (1) feature-based effects of arousal, detailing the results of visual search and attentional blink experiments where the emotional stimuli were task-relevant; (2) spatial effects of arousal, detailing the results of spatial cueing and free viewing task with task-relevant emotional content; and (3) facilitating and hindering effects of arousal of task-irrelevant emotional stimuli (on the main task), detailing the results of the experiments where the emotional stimuli were presented shortly before or during the actual task.

**Table 1:** Summary of included articles

Author	Year	No. of experiment if more than one	Participants	Sex	Type	Stimuli	Arousal measure	Measure of individual differences	Paradigm	Findings
Anderson	2005	Experiment 1	40	N/A	Behavioural	Unpleasant low and highly arousing emotion-laden and neutral words	Rated by independent judges	No	Attentional blink task in rapid serial visual presentation	Detection of negative words were more accurate. The impairing effect of lag was less pronounced for high compared to low arousal negative words
		Experiment 2	36	N/A	Behavioural	Pleasant low and highly arousing and neutral emotion-laden words	Rated by independent judges	No	Attentional blink task in rapid serial visual presentation	Same as in Experiment 1 for negative arousal
		Experiment3A-C	60	N/A	Behavioural	Arousing and nonarousing emotion-laden words, mixed valence	Rated by independent judges	No	Attentional blink task in rapid serial visual presentation	Previous results are unaffected by orthographic distinctiveness, item distinctiveness and unexpectedness
		Experiment 4A-B	40	N/A	Behavioural	Arousing and nonarousing emotion-laden words, mixed valence	Rated by independent judges	No	Attentional blink task in rapid serial visual presentation	The advantage was greater in dual compared to single-task conditions. Enhancement of preattentive bottom-up rather than top-down processing
Ásgeirsson & Nieuwenhuis	2017	Experiment 1A	28	F = 22, M = 16	Behavioural	Pleasant, unpleasant and neutral IAPS pictures	Ratings from IAPS	No	Visual search task with letters as targets preceded by an IAPS picture as prime	No effect of the arousal manipulation
		Experiment 1B	6	F = 5, M = 1	EEG	Pleasant, unpleasant and neutral IAPS pictures	Ratings from IAPS	No	Visual search task with letters as targets preceded by an IAPS picture as prime	Arousing pictures caused significant LLP modulation (400–600 ms) but no behavioural effects
Ásgeirsson & Nieuwenhuis	2019	Experiment 1	29	F = 26, M = 3	Behavioural	Unpleasant and neutral IAPS pictures	Ratings from IAPS	No	Visual search task with letters as targets preceded by an IAPS picture as prime	No effect of the arousal manipulation
		Experiment 2	41	F = 32, M = 9	Behavioural	Unpleasant and neutral IADS sound clips	Ratings from IADS	No	Visual search task with letters as targets preceded by an IADS sound clip as prime	No effect of the arousal manipulation
		Experiment 3A-B	32	F = 25, M = 7	EEG	Unpleasant and neutral IAPS pictures	Ratings from IAPS	No	Visual search task with letters as targets preceded by an IAPS picture as prime	Arousing pictures caused significant LLP modulation (400–600 ms and 800–1200 ms) but no behavioural effects
Astudillo et al	2018		28	F = 14, M = 14	Ocular behaviour & Pupillometry	Pleasant, unpleasant and neutral IAPS pictures	Ratings from IAPS	No	Free viewing of collages of emotional pictures	High arousal compared to low arousal pictures increased dwelling time especially for negative pictures. Pupillometry indicates the effect is mainly due to the arousal level
Fernandez et al	2021		30	F = 21, M = 9	Behavioural	High- and low-arousal instrumental classical music	Rated by subjects in a previous study	Amusia, no change in arousal effect	Attention Network Task	Faster correct target detection during joyful (high arousal) compared to sad (low arousal) music
Heim & Keil	2019		20	F = 15, M = 5	Behavioural & EEG	Low and high arousing IADS sounds clips, mixed valence	Ratings from IADS	Screened for (left) handedness as exclusion criteria	Simple arithmetic operations of two numbers while hearing IADS sound clips as distractors	High-arousing compared to low-arousing sounds were more distracting shown by diminished visuocortical responses and poor task performance
Jefferies et al	2008		100	N/A	Behavioural	Pleasant and unpleasant music with low and high arousal	Participants rated their mood (baseline and five times during the task)	Screened for depression as exclusion criteria	Attentional blink task	No effect for first-target accuracy. Second target accuracy dropped for negative high arousal compared to negative low arousal while positive conditions were in-between these
Keil & Ihssen	2004	Experiment 1	19	F = 11, M = 8	Behavioural	Pleasant and unpleasant emotion-laden words with high arousal and neutral control	Rated by independent sample	No	Attentional blink task	No effect for first-target. Arousal enhanced accuracy for second target, especially for short stimulus onset asynchrony
		Experiment 2	19	F = 12, M = 7	Behavioural	Pleasant and unpleasant emotion-laden words and neutral control—same arousal across categories	Rated by independent sample	No	Attentional blink task	Previous results are not due to emotional valence
		Experiment 3	16	F = 10, M = 6	Behavioural	Pleasant moderate arousal and unpleasant high arousal emotion-laden words and neutral control	Rated by independent sample	No	Attentional blink task	Same as in Experiment 1 for high arousal
Leclerc & Kensinger	2008		48	F = 29, M = 19	Behavioural	Pleasant, unpleasant and neutral pictures	Rated by independent sample	Measured depression, dysexecutive syndrome, anxiety, digit symbol substitution, working memory capacity, arithmetic measures, semantic fluency, vocabulary, mental control, self-ordered pointing, executive control but the effects of these variables were not analysed	Visual search task	High compared to low arousal pictures processed more quickly
Lee et al	2012		20	F = 10, M = 10	Behavioural	Unpleasant and neutral IAPS pictures	Rated by independent sample	No	Visual search task with salient and non-salient tilted lines as targets preceded by an IAPS picture as prime	High compared to low arousing picture enhanced identification of the salient target but impaired identification of the non-salient target
Lee et al	2014	Experiment 1	52	F = 38, M = 14	Behavioural	Arousing and nonarousing sounds, fear conditioning with faces from multiple stimuli libraries	No	No	Dot-probe task	Arousing sounds facilitated RTs for salient-location targets and impaired RTs for non-salient location targets
		Experiment 2	20	F = 11, M = 9	fMRI & behavioural	Arousing and nonarousing sounds, fear conditioning with faces from multiple stimuli libraries	Validated by fMRI results with the difference between BOLD responses to CS + and CS-	No	Dot-probe task	Arousing sounds facilitated RTs. Arousal increased activity in amygdala, face fusiform are and place area
Lundqvist et al	2015		40	F = 20, M = 20	Behavioural	All seven basic emotional facial expressions from the AKDEF pictures	Rated by participants	No	Visual search task	Arousal saliency was in a negative relationship with RTs and positive relationship with accuracy
Lundqvist et al	2014	Cumulative data of seven previous experiments	190	N/A	Behavioural	Angry, happy and neutral facial expressions from the KDEF pictures	Rated by participants	No	Visual search task	Size and direction of differences in detection speed between angry and happy expression follow the corresponding differences between the arousal ratings of the stimuli
Murphy et al	2010	Experiment 2	46	N/A	Behavioural	Low and high arousal IAPS pictures, no information on valence	Rated by participants	Depression, anxiety, general capacity to control attention (ability to focus, shift between tasks, flexibility of control). Negative relationship between ACS scores and overall RT, no other effects achieved significance	Central IAPS image flanked by two outer images, task was to decide whether outer images are same or different	Judgment time was longer on trials when the central image was high compared to low in arousal
Ni et al	2011		22	F = 12, M = 10	Ocular behaviour & Pupillometry	Low, medium and high arousal IAPS pictures, mixed valence	Rated by participants	No	Free viewing of emotional pictures, followed by a control task to ensure participants scanned the images	Low arousal pictures had shorter mean scan path diameters compared to medium and high arousal pictures. Medium arousal pictures elicited the smallest pupil sizes, while low arousal pictures elicited larger pupil sizes compared to high arousal pictures
Sato & Yoshikawa	2010	Experiment2	17	F = 11, M = 6	Behavioural	Angry, happy and neutral facial expressions (Ekman faces) and anti-expressions	No	No	Visual search task	Detection times correlated negatively with experienced emotional arousal
Saito et al	2021	Experiment 2	94	N/A	Behavioural	Neutral faces associated with either monetary reward or punishment or zero outcome	No	No	Visual search task	Reaction times were negatively related to arousal ratings irrespective of valence
Sawada et al	2014a		20	F = 6, M = 14	Behavioural & EEG	Angry, happy and neutral facial expressions (Ekman faces) and anti-expressions	No	No	Visual search task	Higher arousal ratings were related to shorter detection times, more accurate detection and elicited larger EPN (200–400 ms)
Sawada et al	2014b		90	F = 44, M = 46	Behavioural	Angry, happy and neutral facial expressions (Ekman faces) and anti-expressions	No	Screened for (left) handedness as exlusion criteria	Visual search task	Higher arousal ratings were related to shorter detection times. This effect is more pronounced in females compared to males
Sawada & Sato	2015		34	F = 16, M = 18	Behavioural	Angry, happy and neutral facial expressions (Ekman faces) and anti-expressions	No	Screened for (left) handedness as exlusion criteria	Spatial cueing	Higher arousal ratings were related to shorter RTs in the valid trials and longer RTs in the invalid trials
Sawada et al	2016		74	F = 35, M = 39	Behavioural	Angry, happy and neutral facial expressions (Ekman faces) and anti-expressions	No	Screened for (left) handedness as exlusion criteria. Neuroticism was also assessed. The high-neuroticism group showed an overall delay in the detection of target facial expressions and showed higher levels of arousal to facial expressions compared to the low-neuroticism group	Visual search task	Higher arousal ratings were related to shorter detection times in both high- and low-neuroticism participants
Schimmack	2005	Experiment 1	126	F = 63, M = 63	Behavioural	Low, medium and high arousal pleasant and unpleasant IAPS pictures	Rated by independent sample	No	Ignoring emotional pictures while solving simple arithmetic operations of two numbers	More arousing pictures lead to slower problem-solving, but no difference in accuracy
		Experiment 2	60	F = 30, M = 30	Behavioural	Low, medium and high arousal pleasant and unpleasant IAPS pictures	Rated by independent sample	No	Ignoring emotional pictures while detecting the location of a line	More arousing pictures lead to slower problem-solving, but no difference in accuracy
Schupp et al	2007		16	F = 8, M = 8	Behavioural & EEG	Medium and high arousal pleasant and unpleasant IAPS pictures and neutral control	Ratings from IAPS	No	Target counting task—lines superimposed on the pictures	Higher activation and task-interference for arousing compared to neutral stimuli. This was attenuated in highly demanding tasks
Sutherland & Mather	2018		55	F = 39, M = 16	Behavioural	Neutral valence low arousal and pleasant, unpleasant high arousing IADS sounds clips	Rated by participants	No	Visual search task with high and low salience letters as targets preceded by an IADS sound as prime	Arousal increased identification of letter with high but not of low physical saliency
Sutton & Lutz	2019		59	F = 26, M = 33	Behavioural	Positive and negative, low and high arousal emotion-laden words and images from ANEW and IAPS	Ratings from ANEW and IAPS	Screened for depression as exlusion criteria	Dot-probe task	Participants responded faster to stimuli that were high in arousal, congruency effect for the high arousal stimuli was larger. Regarding words, valence effect only. Regarding pictures, arousal effect for positive pictures
Svärd et al	2014		79	F = 45, M = 34	Behavioural	Angry, happy and neutral facial expressions from the AKDEF pictures	Rated by participants	Anxiety and depression but the effects were not analysed	Visual search task	Higher arousal ratings were related to shorter detection times
Turkileri et al	2021		46	F = 38, M = 8	Behavioural & SCR	Fear-conditioned and neutral tones	Participants rated how they felt (baseline and after conditioning), validated by SCR difference between CS + and CS-	No	Visual search task with previously learned locations	Faster target detection for fear-conditioned tones compared to neutral ones
Writh & Kunzmann	2018	Experiment 1	84	N/A	Ocular behaviour	Unpleasant medium and high arousal IAPS pictures	Rated by participants	Education, occupation, self-reported health, life satisfaction, positive–negative affectivity, deductive reasoning, attentional functioning. Statistically controlling for effects did not alter the results	Free viewing and directed attention to neutral or emotional content	Arousal did not affect the outcome
		Experiment 2	56	N/A	Ocular behaviour	Neutral low arousal, unpleasant low and high arousal IAPS pictures	Rated by participants	Education, occupation, self-reported health, life satisfaction, positive–negative affectivity, deductive reasoning, attentional functioning. Statistically controlling for effects did not alter the results	Free viewing and directed attention to neutral or emotional content	Arousal did not affect the outcome
Zsido et al	2018	Experiment 1	117	F = 38, M = 79	Behavioural	Pleasant, unpleasant and neutral IAPS pictures with medium and high arousal	Rated by independent sample	No	Number finding task superimposed on IAPS picture	Low arousal unpleasant pictures impaired performance, high arousal compensated for this effect
		Experiment 2	61	F = 50, M = 11	Behavioural	Unpleasant moderate and high arousal and neutral IAPS pictures	Rated by independent sample	No	Number finding task superimposed on IAPS picture	Same as Experiment 1
		Experiment 3	41	F = 22, M = 19	Behavioural	Unpleasant moderate and high arousal and neutral IAPS pictures	Rated by independent sample	Snake phobia, but no effect was found	Number finding task superimposed on IAPS picture	Same as Experiment 1
Zsido et al	2019		53	F = 32, M = 21	Behavioural	Unpleasant moderate and high arousal IAPS pictures with and without evolutionary relevance	Rated by independent sample	No	Visual search task	High arousal pictures without evolutionary relevance facilitated RTs, with evolutionary relevance hindered RTs
Zsido et al	2020	Experiment 1	53	F = 38, M = 15	Behavioural	Unpleasant moderate and high arousal and neutral IAPS pictures	Rated by participants	Snake phobia, but no effect was found	Number finding task preceded by an IAPS picture	Low arousal unpleasant pictures impaired performance, high arousal compensated for this effect but only for longer presentation times
		Experiment 2	25	F = 16, M = 9	Behavioural	Unpleasant moderate and high arousal and neutral IAPS pictures	Rated by participants in Exp1	No	Number finding task preceded by an IAPS picture	Same as Experiment 1
Zsido et al	2021		44	F = 30, M = 14	Behavioural & eye-tracking	Unpleasant moderate and high arousal and neutral IAPS pictures	Rated by independent sample in previous study	Screened for anxiety as axclusion criteria	Number finding task with task-irrelevant emotional stimuli outside of central vision	Low arousal unpleasant pictures impaired performance, high arousal compensated for this effect but only for longer presentation times. Participants fixated threatening pictures later and for shorter durations compared to neutral images

Stimuli column identifies the type and modality of emotional stimuli used. Paradigm column denotes the type of task used

*F* female, *M* Male, *N/A* no data reported, *IAPS* International Affective Picture System, *IADS* International Affective Digital Sounds, *ANEW* Affective Norms for English Words, *KDEF* Karolinska Directed Emotional Faces, *EPN* early posterior negativity, *LLP* late positive potential, *RTs* reaction times

### Feature-based effects of task-relevant arousal

A total of 11 *visual search studies* used task-relevant emotional stimuli. Eight of these used facial expressions (Lundqvist et al., 2014, 2015; Saito et al., 2021; Sato & Yoshikawa, 2010; Sawada et al., 2014a, b, 2016; Svärd et al., 2014), two used IAPS pictures (Leclerc & Kensinger, 2008; Zsido et al., 2019a), and one used auditorial stimuli (Turkileri et al., 2021). The unequivocal consensus of these experiments is that RTs were shorter for finding stimuli that had higher arousal ratings.

Two studies (Lundqvist et al., 2015; Sawada et al., 2014b) also point out that higher arousal resulted in higher accuracy in finding the target. Further, in one study Lundqvist et al., (2014) demonstrate that the arousal dimension is important in categorical emotion theory-based experiments and picture databases similar to their dimensional emotion theory-based counterparts. One study (Zsido et al., 2019a) only found the arousal effect for modern threatening stimuli (e.g. guns), while they also reported an inverse effect (i.e., higher RTs for higher arousal) for evolutionary relevant threatening stimuli (e.g. snakes). Regarding physiological results, one EEG study (Sawada et al., 2014b) noted that higher arousal elicited larger early posterior negativity (EPN, 200–400 ms). As for individual differences, Sawada and colleagues observed that the arousal effect was more pronounced in female compared to male participants^2^ (Sawada et al., 2014a), but found no difference in participants with low compared to high neuroticism (Sawada et al., 2016).

I found two studies (Anderson, 2005; Keil & Ihssen, 2004) with a total of seven experiments using an *attentional blink task* with task-relevant emotional stimuli (words in all cases). In general, higher arousal seems to increase the accuracy of target detection. However, Keil and Ihssen (2004) found no arousal effect for the detection of the first target, only for the second one. Both studies reported that the impairing effect of lag was less pronounced for high compared to low arousal stimuli. Further, while Anderson (2005) found this effect only for negative but not for positive valenced stimuli, Keil and Ihssen (2004) found that their results were not dependent on the valence of the stimuli. Moreover, Anderson (2005) found that the results were unaffected by orthographic distinctiveness, item distinctiveness, and unexpectedness; and that the advantage was greater in dual compared to single-task conditions.

### Spatial effects of task-relevant arousal

All three *spatial cueing studies* used task-relevant emotional stimuli. One study (Lee et al., 2014) used sounds in two experiments, one study (Sawada & Sato, 2015) used facial expressions, and one study (Sutton & Lutz, 2019) used words and IAPS pictures. All four experiments found that higher arousal ratings were related to shorter RTs in the valid trials and longer RTs in the invalid trials. However, Sutton and Lutz (2019) found an arousal effect only for positive pictures, and no arousal effect regarding words. Regarding the neural background, Lee et al. (2014) found that arousal increased activity in the amygdala, face fusiform area, and place area in the second (fMRI-based) experiment.

I found three studies (Astudillo et al., 2018; Ni et al., 2011; Wirth & Kunzmann, 2018) that recorded ocular behavior during *free viewing* of emotional pictures. Astudillo et al. (2018) reported that dwelling time increased for high compared to low arousal pictures. Although this effect was more pronounced when the emotional content was negative, pupillometry indicated that changes in dwelling time were mainly due to the arousal level. Ni et al. (2011) found shorter mean scan path diameters for low compared to medium and high arousal pictures. Further, medium arousal pictures elicited the smallest pupil sizes, while low arousal pictures elicited larger pupil sizes compared to high arousal pictures. In contrast to these, Writh and Kunzmann (2018) found that the arousal of the pictures used did not affect the outcome of their results in the two experiments they conducted.

### The effects of task-irrelevant arousal

I found five studies where the task-irrelevant emotional *stimuli preceded the presentation of the task*, in all cases visual search paradigms were used. Four studies used visual stimuli (IAPS pictures) to elicit emotional arousal (Ásgeirsson & Nieuwenhuis, 2017, 2019; Lee et al., 2012; Zsido et al., 2019b). Auditory stimuli were used in one study (Sutherland & Mather, 2018) and one experiment (Ásgeirsson & Nieuwenhuis, 2019). Three studies (Lee et al., 2012; Sutherland & Mather, 2018; Zsido et al., 2019b) found that high compared to low arousing prime stimuli increased visual search performance, i.e. target identification was faster. Zsido et al. (2019b) also showed that this effect persisted over time, i.e. performance was better even if the task comprised multiple targets (numbers) that had to be found in ascending order. Based on the results of Lee et al. (2012) and Sutherland and Mather (2018) arousal only has a facilitating effect for targets with high but not low physical salience. In contrast, Ásgeirsson and Nieuwenhuis (2017, 2019) found no behavioral effects of the arousal manipulation across five experiments, although arousing pictures caused significant LLP modulation (400–600 ms and 800–1200 ms) per the EEG results.

There were seven studies where task-irrelevant emotional *stimuli were presented during the task* (Fernandez et al., 2021; Heim & Keil, 2019; Murphy et al., 2010; Schimmack, 2005; Schupp et al., 2007; Zsido et al., 2018, Zsidó et al., 2022). These studies were diverse regarding both the task (ANT, solving arithmetic operations, matching, target counting, and visual search) and the type of stimuli (classical music, sounds, and pictures) they used. Studies (Heim & Keil, 2019; Murphy et al., 2010; Schimmack, 2005; Schupp et al., 2007; Zsido et al., 2018, Zsidó et al., 2022) that used emotional stimuli as distractors found that arousal can impair task performance. However, one study inducing emotional arousal with background music (Fernandez et al., 2021) found that subjects detected targets faster whit higher emotional arousal. Consistent with this, Zsidó et al. (2022) found that although less arousing unpleasant pictures hindered, highly arousing pictures increased visual search performance. Schupp et al. (2007) reported that arousal only interfered in the easy and not in the hard task condition, a pattern that was supported by EEG evidence. That is, arousing stimuli produced higher activation (increased EPN approx. 150–300 ms post-stimulus) in the easy compared to the hard task condition. Somewhat in line with this, Heim and Keil (2019) observed that high compared to less arousing and neutral sounds resulted in diminished steady-state visual evoked potentials (ssVEPs) both in the early (400–2600 ms) and late (3400–5600 ms) time windows, meaning that the task interference was greater for high compared to less arousing stimuli. Taken together with the eye-tracking results of one study (Zsidó et al., 2022), where participants fixated on threatening pictures later and for shorter durations compared to neutral images, it seems plausible that highly arousing images attract covert rather than overt eye movements.

## Discussion

Emotional arousal received relatively little attention in the past two decades compared to its equally important counterpart, emotional valence. The aim of the present paper was threefold. First, to fill a gap and systematically review the effects of emotional arousal on visual attentional performance as previous reviews were mostly concerned with the effects of the valence of emotionally charged stimuli (i.e. positive and negative) on visual processing (Gerdes et al., 2014; Olofsson et al., 2008). Second, to clarify how existing arousal theories can explain the effects found on visual attention under various conditions. I sought to examine the effects of task-relevant and task-irrelevant arousing stimuli across various modalities and paradigms relying on behavioral, eye-tracking, and neurological measures. Third, to raise awareness of the fact that emotional arousal (besides and regardless of valence) should be investigated on its own terms. Dimensional theories of emotions propose that valence and arousal are independent dimensions (Bliss-Moreau et al., 2020; Sutherland & Mather, 2018) which is rarely the case in experimental studies (Dan-Glauser & Scherer, 2011; Deák et al., 2010; Lang et al., 1997; Pool et al., 2016). Instead, the two dimensions overlap, and distinguishing their unique effect is often hard (Yiend, 2010). Studies dealing with the effect of one emotional dimension have limited options to account for this. The solution is either to collapse studies across arousal levels (e.g., from low to high) or to control for the level of arousal (e.g., using only moderately arousing stimuli). Here I took a mixed approach, that is, to collapse all studies that examined arousal irrespective of the valence of the stimuli but note valence-dependent associations where possible.

The number of studies found and retained showed that while a lot of papers mention emotional arousal (123), only a handful of these (32, i.e., 26%) systematically addressed how it affects visual search performance. Studies that were not included in this review were rejected mostly for two reasons: (1) they assessed the arousal of the stimuli but did not use it in the analyses or (2) they used the terms interchangeably. The problem is, even if these studies did manipulate arousal, for instance by comparing positively and negatively valenced stimuli that have similar arousal ratings to neutral stimuli, it is impossible to disentangle the effects caused by the arousal of the stimuli used. A reexamination of existing data, similar to a previous study (Lundqvist et al., 2014), would be a good approach to clarify the unique roles of the two emotional dimensions. Further, in future studies, it is important to also report results regarding the arousal level (even null findings) and to make sure that terminology is used in a precise and uniform manner in an effort to make the results comparable to that of other studies.

Overall it seems that the way emotional arousal affects visual search performance is mostly defined by the task relevance of the stimuli that convey the emotion. The findings of this review suggest that task-relevant arousing stimuli (regardless of the modality) draw (evident from e.g., visual search studies) and hold attention (as demonstrated by e.g., free viewing studies), resulting in shorter RTs and increased accuracy. Since such highly salient objects are processed before other stimuli (Mather & Sutherland, 2011; Mather et al., 2016) and a greater capacity is devoted to them (Kahneman, 1973), their evaluation, and consequently, movement initiation happens earlier (Csathó et al., 2008; LeDoux & Daw, 2018; Öhman, 2005). In contrast, when the arousing stimuli are task-irrelevant, the results are mixed and dependent on the presentation and modality used (i.e., visual or auditory) and the stimulus onset properties (i.e., whether the task-irrelevant stimulus precedes the visual attention task, or if it is presented at the same time). When the emotional content precedes the task or music is used during the task, or the content is presented for a long duration (because solving the task takes several seconds), arousal may also increase visual attentional performance. This is in line with previous findings on threatening stimuli (Cohen et al., 2011; O’Toole et al., 2011) and that valence-based attentional biases vary as a function of arousal (Pool et al., 2016). Highly arousing stimuli can increase the available cognitive (working memory) capacity (Nielson et al., 1996; Sakaki et al., 2019) and make attentional movements faster (Bradley et al., 2011; Gomez et al., 2019). When presented as task-irrelevant distractors during the primary task, arousing stimuli impaired task performance. Based on the theories that propose a competition for the limited working memory resources, it seems plausible to claim that the highly arousing stimulus won the competition, and the active inhibition of its processing (and disengagement from it) is necessary to complete the task (Burra et al., 2019; Trujillo et al., 2021). The active suppression of emotional stimuli is harder compared to neutral stimuli, and the cognitive capacity to maintain the suppression throughout the task could be proportional to the arousal the emotional stimuli elicit (Hindi Attar et al., 2010).

While all current arousal theories (Kahneman, 1973; Mather et al., 2016; Mather & Sutherland, 2011; Zsido et al., 2019b) are capable of explaining these results, I found no decisive evidence of whether the arousal-induced increase in capacity is real or just apparent, and how to best explain this phenomenon. A possible explanation behind the mixed result could be the impact of appraisal in processing emotional stimuli (Pool et al., 2016). Such appraisals include the goal relevance, goal congruence, and novelty of the emotion, as well as the perceived agency, and the control or coping potential of the perceiver (Sander et al., 2018). Appraisal theories emphasize the role of individual differences positing that the level of arousal elicited by a particular stimulus can greatly differ between participants in a study (Pool et al., 2016; Sander et al., 2005). The studies presented in this review did not seek to test these aspects of the processing. Further, the objective physiological state of arousal elicited by the stimuli used during the experiment was not measured in most cases. While the emotional stimuli used in such studies are standardized and often participants provided a subjective rating of it after the experiment, these ratings were only used to underscore that emotional stimuli did differ from neutral control stimuli but were not used in the statistical analysis. That is, the variance (or noise in the present case) in data introduced by individual differences in the appraisal was not controlled for. Separating this relevance effect from the arousal effect is difficult, although appraisal theories only posit that stimuli appraised affectively relevant have facilitated access to attention (Brosch et al., 2013; Sander et al., 2003). This is not contradicted by arousal theories arguing that all emotional stimuli could increase arousal (Brosch & Sharma, 2005; Mather et al., 2016; Sutherland & Mather, 2018). Consequently, the fact that often mixed and a wide variety of stimuli were used (positive–negative, erotic-threatening, etc.) to achieve the increase in arousal may be a strength of the studies insofar as it reduces the effect of appraisal and increases the effect of (general) arousal. The inconsistencies in the results could be mended by future research experimentally and systematically testing the various modalities, presentation types, methods used, and the role of appraisal. Similarly, individual differences regarding current mood, anxiety, depression, or the sensitivity of the approach-avoidance system could also play a role in the effect of arousal on visual search performance. Individual differences along these variables are well-documented concerning emotion processing (Fraga et al., 2021; Mueller & Kuchinke, 2016) and attentional bias for threat framework (Koster et al., 2006; McNally, 2018). Yet, the studies in the current review did not analyze such effects. Introducing emotional arousal as a manipulated variable in tasks adds a significant amount of understanding to attentional biases to emotional stimuli, and the possible hindering or facilitating effects of the presence of emotional arousal on task performance. Such studies provided evidence that arousal may have effects independent of valence, and that there is an extended neurological basis for attentional bias toward emotionally charged stimuli. Examining the impact of arousal and valence on visual attention in larger samples would be helpful.

Although this review offered a detailed comparison between the studies included in the literature, there were some limitations. Only studies that investigated visual attentional performance were included, which may not lead to a comprehensive understanding of attentional bias toward emotional arousal and changes in performance elicited by arousing stimuli. I did not analyze effect sizes because of the small amount of similarity between studies. This includes differences in the modality of the stimuli, task design, and the paradigm used. Conducting a more specific search would have resulted in a too-low number of eligible papers. Further, although the anxiety level-dependent changes in attention for negative arousal (i.e., threat) are well-documented (Burra et al., 2019; Kim et al., 2021; Lee et al., 2014; March et al., 2017; Mather & Knight, 2006; Yiend, 2010), I have decided to take a more inclusive approach and focus on arousal in general, regardless of other effects. My conclusions might only apply to non-clinical populations, as the emotional stimuli are processed differently in clinical compared to non-clinical populations (Yiend, 2010). Even with these limitations, the present review highlights the necessity for future research targeting the role and underlying mechanisms of emotional arousal on attentional processes.

Future studies should also focus on the potential effects of individual differences due to the appraisal of the stimuli used and the current mood of the respondent or other personality-related measures (such as anxiety, depression, and the sensitivity of the approach-avoidance system) as there might be great differences in emotion processing along these dimensions (Fraga et al., 2021; Mueller & Kuchinke, 2016). Further, the effects of non-emotional arousal should also be considered, as such stimuli could help separate arousal from other emotional dimensions (especially valence) and these experiments may have further practical relevance. Indeed, by studying cognition and action, the role of arousal is introduced to a completely new branch of research, and the practical effects of how arousal affects visual cognition could be examined.

## Data Availability

Data sharing not applicable to this article as no datasets were generated or analysed during the current study.

## Footnotes

1 The gender ratio was not disclosed in 11 experiments.

2 The authors suggest based on the result of previous studies that this might be associated with the fact that females tend to show a slight advantage in the recognition of emotional facial expressions compared to males.
